# Laryngeal cartilage calcifications on lateral cephalometric radiographs

**DOI:** 10.1038/s41598-024-52968-7

**Published:** 2024-01-29

**Authors:** Magdalena Sycińska-Dziarnowska, Steven J. Lindauer, Liliana Szyszka-Sommerfeld, Gianrico Spagnuolo, Krzysztof Woźniak

**Affiliations:** 1 Department of Orthodontics, Pomeranian Medical University in Szczecin, Al. Powst. Wlkp. 72, 70111 Szczecin, Poland; 2 Department of Orthodontics, School of Dentistry, Virginia Commonwealth University, Richmond, VA 23298 USA; 3 Department of Neurosciences, Reproductive and Odontostomatological Sciences, University of Naples “Federico II”, 80131 Napoli, Italy

**Keywords:** Medical research, Signs and symptoms

## Abstract

The aim of this study was to determine the influence of age and gender on the incidence of calcification in laryngeal cartilage diagnosed on lateral cephalometric radiographs routinely taken for orthodontic diagnosis. The lateral cephalometric radiographs of 957 patients who met the study criteria were analyzed from among the 1000 lateral radiographs originally collected. The images were evaluated independently by two investigators. Given the dichotomous dependent variable (calcification or no calcification), a mixed logistic regression model was used to test how age and gender affected calcification. The effect of age and gender reliably determined the likelihood of laryngeal cartilage calcification. The greatest differences in the degree of calcification by gender were found at ages 20–25 years. The degree of calcification increased with age, reaching 100% in women at age 30 and in men at age 50. In women, the degree of calcification was higher than in men from the age of 13 years and levelled off at the age of 50 years. The interrater agreement was strong *k* = 0.97, *z* = 30.0, *p* < .001. Calcification can be detected by orthodontists trained in lateral cephalogram analysis and can be used as a screening or diagnostic tool to detect calcified areas in the larynx.

## Introduction

Cartilage forms the skeleton of the larynx and gives it structure. The cricoid cartilage forms the base of the larynx and is the complete ring. Above the cricoid cartilage, connected to its lateral edges, is the shield-like thyroid cartilage. This cartilage provides protection for the internal components of the larynx ^1^. The thyroid cartilage is the largest cartilage of the larynx and is located between the vertebral levels C4-C5. It is formed by two laminae of hyaline cartilage that meet in the midline anteriorly to form an angle, called the Adam’s apple ^2,3^.The posterior part is extended upward and downward as a cornu. The cricoid cartilage is located at C6 below the thyroid cartilage and articulates on its inferior cornu ^2,3^.

As part of the aging process, calcification and ossification occur in various cartilaginous structures of the laryngeal skeleton, which is primarily composed of hyaline cartilage. The literature indicates that this process begins in the third decade of life ^4^. Ossification and calcification of laryngeal cartilage has been the subject of research since Chievitz’s anatomical study in 1882 ^5^. He found that ossification usually begins after the completion of skeletal growth at the age of 20 years in men and 22 years in women ^4,5^. Zan et al. classified the mineralization process as calcification, sclerosis, and ossification ^6^. The terms “ossified” and “calcified” can be used synonymously, but calcification is the first stage and occurs before ossification, when cartilage turns into bone ^4,7^.

The discovery of x-rays inspired novel research and in 1902 Scheier examined 120 cases histologically and took radiographs of removed cartilage. In 1914, Iglauer was the first to describe the sites and sequence of ossification and he emphasized the value of radiology in living subjects ^7^. Lateral cephalometric radiographs show the relationships between soft tissue and bony landmarks and are used as a standard part of orthodontic diagnosis of facial growth before orthodontic treatment, during treatment to evaluate progress and changes and, at the end of treatment, to check whether goals have been met. In order to obtain standardized and comparable craniofacial images, the radiographs are taken in a cephalostat, which was introduced in 1931 by Broadbent in the US ^8,9^. Radiology has been recognized as an accurate method for detecting calcification and ossification ^10^. Ossification of the thyroid cartilage can be detected on a routine lateral cephalometric radiograph ^11,12^.The increasing number of orthodontic treatments and lateral cephalometric radiographs taken before and during orthodontic treatment provides a large database for the study of laryngeal cartilage changes.

In recent years, there has been a surge in interest in employing advanced imaging modalities. Techniques such as magnetic resonance imaging (MRI) offer a non-invasive and detailed insight into cartilaginous structures, providing an opportunity to distinguish calcification patterns with high precision. This novel approach could potentially complement traditional radiographic methods, offering a comprehensive view of laryngeal region, however it is not routinely taken before orthodontic treatment. Our focus on cephalograms aligns with the wealth of existing data in orthodontic databases, offering valuable insights without necessitating additional imaging procedures.

Given the paucity of studies in this area, the aim of this study was to examine the visible changes on routinely taken lateral cephalometric radiographs, and to determine the influence of age and gender factors on the incidence of calcification in laryngeal cartilage diagnosed on radiographs. The purpose is to alert orthodontists to also routinely evaluate radiographs in the laryngeal region when analyzing cephalometric radiographs.

## Methods

A retrospective study was performed on lateral cephalometric radiographs routinely taken before orthodontic treatment at the Department of Orthodontics at Pomeranian Medical University in Szczecin, Poland. The study was exempted from ethical approval by the Ethical Committee of the Pomeranian Medical University in Szczecin, Poland (declaration reference no. = KB-012/104/09/2021/Z) because the study was based on pre-existing data from radiological records and the research was conducted anonymously. The need to obtain informed consent was waived by the Ethical Committee of the Pomeranian Medical University in Szczecin, Poland (declaration reference no. = KB-012/104/09/2021/Z). Cephalograms were taken with the CRANEX™ 3Dx (Soredex, Kavo Imaging GmbH, Berlin, Germany) at the Department of Radiology at Pomeranian Medical University in Szczecin, Poland in the 5-year timeframe 2018–2022. The lateral cephalometric radiographs of 957 patients who met the study criteria were analyzed from among the 1000 lateral radiographs originally collected for analysis due to the random number selection. The inclusion criteria for the study were: good visibility of laryngeal cartilage and the laryngeal region, and recorded date of birth and date of radiologic examination available. Forty three radiographs were excluded from the study due to not meeting the inclusion criteria. The X-ray images should provide a clear and precise view of the vertebrae (C4-C6) and nearby anatomical structures. This condition is crucial for the effective identification, analysis, and interpretation of anatomical structures, including potential calcifications, the intensity and distribution of radiopaque areas or other pathological changes.

The collected images were evaluated independently by two investigators, orthodontic specialists (M.S.-D. and L.S.-S.) with 10 and 15 years of experience, respectively, in evaluating lateral cephalometric radiographs. Consensus was reached in case of ambiguity. The Cohen's Kappa was used as an index of interrater agreement between two raters on categorical data. Relationships between two categorical variables were estimated using Pearson Chi-square test.

Given the dichotomous dependent variable (calcification or no calcification), a mixed logistic regression model was used to test the research hypothesis. Three working models were estimated: 1) a model with two predictors (sex, age) without interaction, 2) a model with interaction with non-informative priors, $\mathcal{N}\left(0, 10\right)$, and 3) a model with interaction together with informative priors $\mathcal{N}\left(0, 1.5\right)$.

The selection of the final model was based on a comparison of the working models based on the procedure of efficient approximate leave-one-out cross-validation (LOO), (Vehtari et.al, 2017) using Watanabe-Akaike information criterion. The presence of an interaction of predictors along with noninformative priors allowed us to obtain the smallest values for estimating the difference in expected predictive accuracy and standard error and, therefore, this model was used as the final model.

Estimates were performed in the R package brms (Bürkner, 2017) with Stan (Carpenter et.al, 2017) on the backend using Markov Chain Monte Carlo (MCMC) sampling via adaptive Hamiltonian Monte Carlo (Hoffman and Gelman 2014; Stan Development Team 2019). Model coefficients of determination were interpreted by Cohen convention (Cohen, 1988). The value of the target average acceptance probability was set to a conservative value of 0.99, which is more robust to posterior distributions with strong curvature.

The sex categorical variable was coded using the dummy method (with the first category as the reference).

Following the Sequential Effect eXistence and sIgnificance Testing (SEXIT) framework (Makowski et. al, 2019) the median of the posterior distribution and its *95% CI* (Highest Density Interval) were reported, along the probability of direction (pd), the probability of significance and the probability of being large. The thresholds beyond which the effect were considered as significant (i.e., non-negligible) and large were |0.09| and |0.54|.

The convergence of Bayesian sampling was assessed using *R-hat*, which should be less than 1.01. In addition, the stability of Bayesian sampling was assessed using the effective sample size (*ESS*), which should be greater than 1000. Analyses were conducted using the R Statistical language (version 4.2.1; R Core Team, 2022).

### A Ethical approval and consent to participate

The study was exempted from ethical approval by the Ethical Committee of the Pomeranian Medical University in Szczecin, Poland (declaration reference no. = KB-012/104/09/2021/Z). The need to obtain informed consent was waived by the Ethical Committee of the Pomeranian Medical University in Szczecin, Poland (declaration reference no. = KB-012/104/09/2021/Z). All methods were carried out in accordance with relevant guidelines and regulations.

## Results

Lateral cephalometric radiographs of 957 patients (380 males and 577 females; aged 6 to 66 years) taken before orthodontic treatment were used to investigate the presence of calcification in the laryngeal cartilage based on a randomized number selection process. The interrater agreement was strong *k* = 0.97, *z* = 30.0, *p* < 0.001. The characteristics of the study sample are shown in Table 1.

**Table 1:** The characteristics of the sample.

Parameter	Frequency (%)
Sex: - Female - Male	577 (60.3%) 380 (39.7%)
Age group: - 6–20 years - 21–35 years - 36–50 years - 51–66 years	695 (72.6%) 180 (18.8%) 67 (7%) 15 (1.6%)
Calcification: - No - Yes	595 (62.2%) 362 (37.8%)

*Numerical variable was grouped to show the density of the age distribution

**Figure 1:**
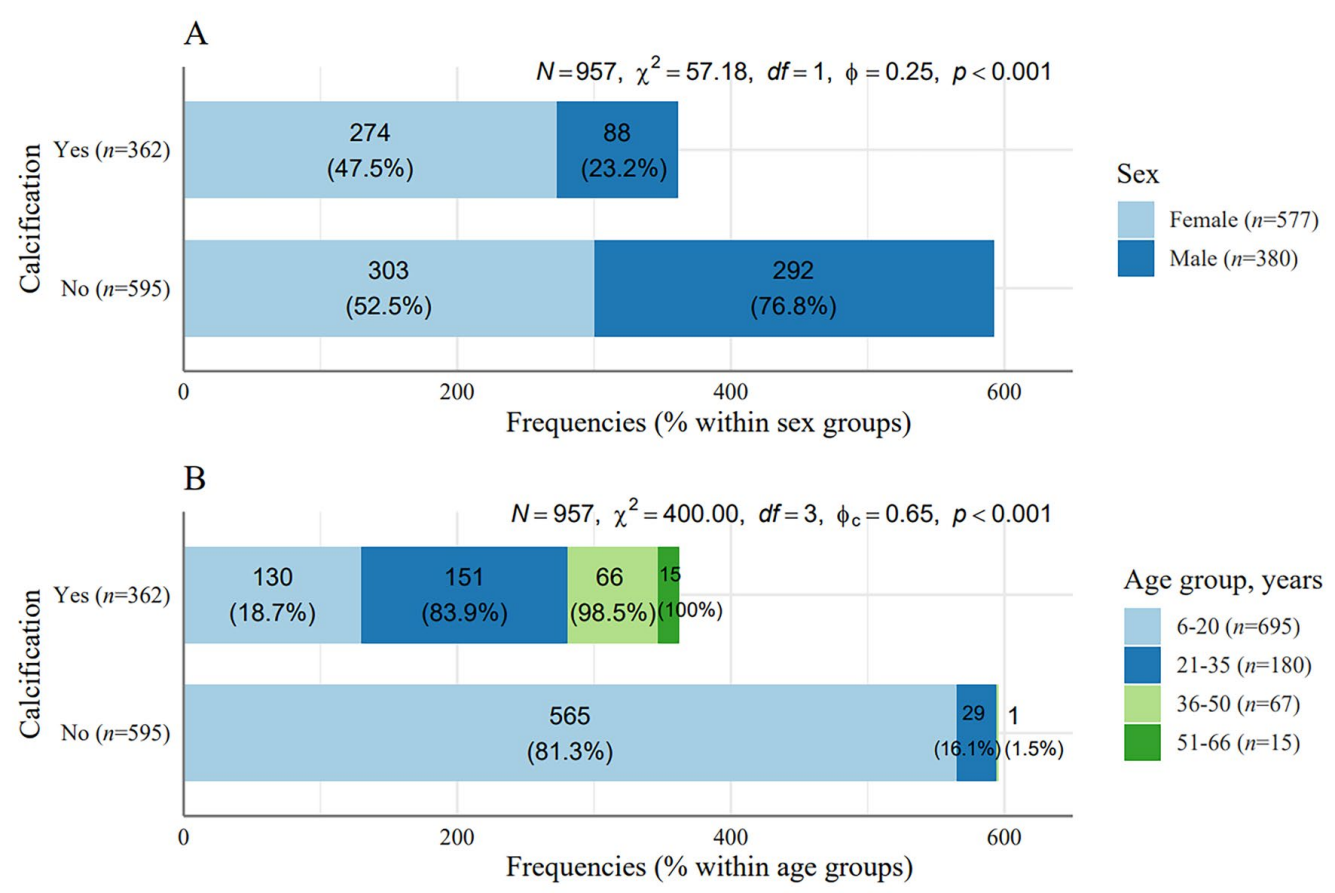
Frequencies and percentages of the occurrence of calcifications in the laryngeal cartilage. * Within sex groups (**A**) and within age groups (**B**) with results of the Pearson Chi-square test (χ^2^). *N*—sample size, *n*- group sample, *df*—degrees of freedom, *p*—*p*-value of statistical test, *φ*—measure of relationship strength Phi, *φ*_*c*_—measure of relationship strength Cramer’s V.

**Figure 2:**
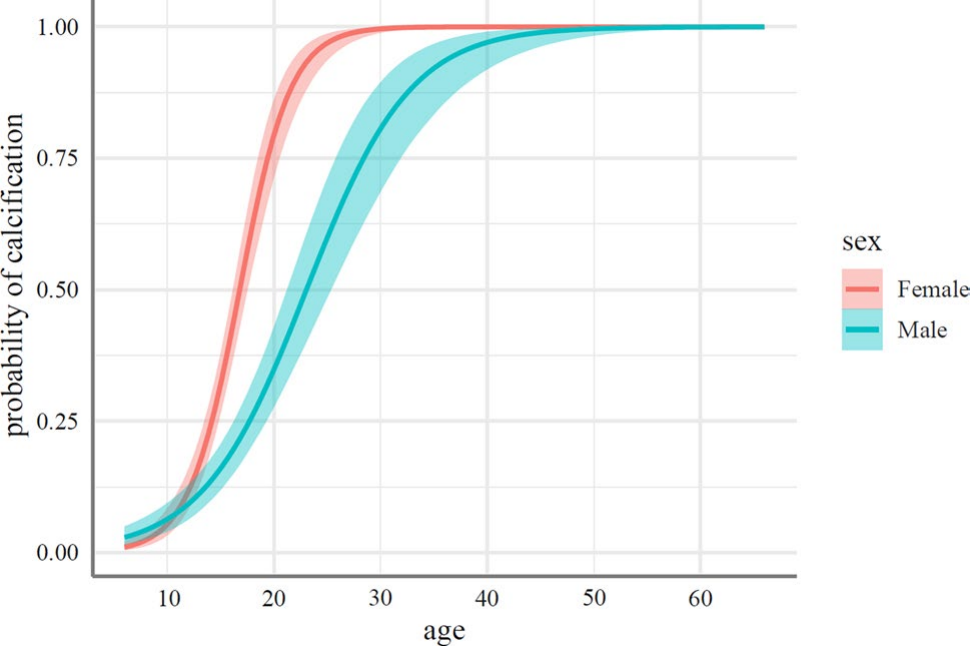
Expected probabilities of calcification in the laryngeal cartilage as a function of sex and age.

A graphical representation of the occurrence of calcification in gender and age groups is shown in Fig. 1. Comparative images illustrating calcification and non-calcification are presented in Fig. 2.

We fit a Bayesian logistic mixed model to predict the probability of calcification based on the predictors of sex [female, male] and age [6–66, xϵ ℕ] ℕ space of natural values and random effects.

The explanatory power of the model was substantial for unadjusted values. *R*^*2*^_*bayes*_ = 0.58, *95% CI* [0.56, 0.60] and adjusted (computed using a leave-one-out-adjusted posterior distribution) *R*^*2*^_*conditional*_ = 0.58, *95% CI* [0.53, 0.63] Bayesian coefficient of determination.

The adjusted population level effects are shown in Table 2.

**Table 2:** Fitted population-level effects, N = 957.

	Estimate	EE*	l-95%CI**	u-95%CI***	R_hat_****	Bull ESS*****	Tail ESS*****
Intercept	−7.09	0.63	−8.41	−5.90	1.001	1913	2438
Sex [Male]	2.32	0.78	0.83	3.90	1.001	1719	2537
Age	0.42	0.04	0.35	0.51	1.001	1781	2131
Sex [Male]: age	−0.22	0.05	−0.31	−0.12	1.001	1644	2177

*EE—estimation error, **l–95% CI—lower level of 95% credible interval, ***u-95%CI—upper level of 95% credible interval, ****R_hat_—the potential scale reduction factor on split chains, *****ESS—effective sample size.

The estimation of all model coefficients were successfully converged (*R*_*hat*_ = 1.00) and were reliable (*ESS* > 1000).

The log *OR* effect (-7.09) of the model intercept (further – the baseline), laryngeal cartilage calcification in women aged 0 years, was negative (100.00% probability), reliable (100.00% probability), and large (100% probability).

The log *OR* effect of calcification was reliably higher (2.32) in men group compared with baseline. The log *OR* effect was positive (99.99% probability), reliable (99.95% probability), and large (99.17% probability).

The log *OR* of calcification was reliably higher (0.42) for each additional patients age year compared to the baseline. The log *OR* effect was positive (100.00% probability), reliable (100.00% probability), and less than large (99.66% probability).

The log *OR* of interaction between age and sex was reliably lower (-0.22) in men compared to the baseline for each additional patients age. The log *OR* effect was positive (100.00% probability), reliable (99.84% probability), and less than large (100.00% probability).

A graphic representation of population-level effects in the form of calcification probabilities is shown in Fig. 2.

Probability values of Fig. 2 for age and sex terms included in Table 3. The solid lines in Fig. 2 represented the median estimated probabilities, serving as an indicator of the central tendency within the 95% credible intervals (*CI*s). Shaded areas around these lines corresponded to the 95% *CI*s, indicating the range of probabilities within which we are 95% confident that the true value lies, providing a visual representation of the uncertainty associated with the estimates. Each sex was denoted by a distinctive color, facilitating a clear comparison between male and female trends as age progressed. The credible intervals offered insight into the precision of our estimates, with narrower intervals suggesting greater certainty.

**Table 3:** The estimated marginal medians (predicted values) for the response variable for sex and age terms.

Model terms		Estimate*	SE**	L–95% CI	u—95% CI
Age	Sex				
10	Female	0.06	0.01	0.04	0.09
	Male	0.07	0.01	0.04	0.10
15	Female	0.33	0.03	0.27	0.39
	Male	0.16	0.02	0.12	0.21
20	Female	0.82	0.04	0.75	0.89
	Male	0.37	0.04	0.30	0.46
25	Female	0.97	0.01	0.94	0.99
	Male	0.62	0.06	0.50	0.73
30	Female	1.00	0.01	0.99	1.00
	Male	0.81	0.05	0.69	0.90
35	Female	1.00	0.01	0.99	1.00
	Male	0.92	0.03	0.84	0.97
40	Female	1.00	0.01	1.00	1.00
	Male	0.97	0.01	0.93	0.99
50	Female	1.00	0.01	1.00	1.00
	Male	1.00	0.01	0.98	1.00

*Probability of calcification.

**SE—the standard error.

Table 3 includes age and sex, providing a detailed breakdown across different age groups and between male and female subjects. Key observations from the table: Age Trends: The estimated probability of calcification generally increases with age for both female and male subjects. Sex Differences: There are variations in the estimated probabilities between male and female subjects within the same age group. For instance, at age 10, females have a lower estimated probability compared to males, but this trend reverses as age increases. These findings suggest an age-dependent and sex-specific pattern in the occurrence of calcification, providing valuable insights into the factors influencing laryngeal cartilage calcification based on the analyzed variables.

## Discussion

The general consensus in the literature is that the laryngeal cartilage ossifies gradually with age as a physiological process ^13,14^. The radiographically visible ossification of laryngeal cartilage increases with age, beginning in the third decade of life ^12,15^. Increasing ossification with age is consistent with the results of our examination of 957 cephalometric radiographs, in which calcification was observed on all lateral cephalometric radiographs in women older than 30 and in men older than 50, but was also detected in younger subjects, a finding not widely reported in previous studies. In previous studies, the incidence of laryngeal cartilage ossification among men was higher than in women ^12,14,16^.There was no concordance between our study according to gender; the incidence of calcification in our study was higher in the female group. In contrast, a study by Glikson et al. found a higher incidence of calcification in female groups, except for the age group of 20 years ^17^.

Mupparapu and Vuppalapati reported ossification of the laryngeal cartilage in a 14-year-old boy as an atypical early presentation ^11^. According to the authors, such changes are visible only in individuals over 20 years of age, and it was unusual to detect ossification in children or adolescents ^11^. Upper respiratory tract calcification is unique in people under the age of 15 ^18^. In contrast, in our study, calcification was detected in 130 cases in the 6–20 age group with a female predominance. In contrast, according to the ultrasound results, the ossification process of the thyroid cartilage begins in the first decade of life. In a group of 419 children, ossification foci were found in 167 children and increased with age in both sexes. The first ossification was detected in a 72-month-old girl. Radiologists should be aware of this issue to avoid misdiagnosing them as pathological masses ^19^.

Many authors have conducted studies on the ossification process of laryngeal cartilage using radiographs to estimate the age of individuals. However, it does not seem possible to estimate age based on ossification observed in radiographs due to the high degree of variability, as observed in the study by Garvin et al. ^16^. Also, Dang-Tran et al. stated that methods based on ossification of the laryngeal cartilage are not accurate enough to estimate the age of individuals based on the degree of calcification ^20^. Data mining technology was supposed to improve the accuracy of adult age estimation but, in practice, the accuracy of adult age estimation based on laryngeal cartilage ossification has a large error of 8.6 years for males and 12.6 years for females, so it should be combined with other age indicators ^21^. Also in our study, calcification in adolescents showed different inter-individual variability.

Ossification can be considered physiologic when there are no clinical features of underlying systemic disorders ^11^. Asymmetric mineralization of the thyroid cartilages was present in 12.9% of cases in 650 cervical CT scans. This should be taken into account when evaluating CT scans of patients with laryngeal cancer to avoid false-positive results ^6^. Metastasis to the larynx is rare because the cartilaginous tissue lacks vessels. Metastatic calcification is associated with dysregulation of serum calcium and phosphorus levels and primarily affects the arteries and visceral organs ^22^. The probability of metastasis increases with age, and applies to melanoma, renal cell carcinoma and prostate cancer. Annovazzi et al. described a case of an over-80-year-old man with prostate cancer and metastasis to the thyroid cartilage as the only site of recurrence ^23^. Laryngopharyngeal carcinoma is often due to tumor infiltration into the cartilage at a higher grade of cancer ^24^. A 55-year-old man from Japan was diagnosed with osteosarcoma of the larynx, which was characterized by a high degree of calcification and enlargement of the thyroid cartilage. Sarcomas of the larynx are rare tumors ^25^. Although they are rare, orthodontists who may evaluate the laryngeal region on radiographs in daily practice should be aware of these findings. In unclear cases, a family doctor should be consulted to rule out parathyroid hormone-level or calcium-phosphate disorders.

Another interesting point is that diffuse calcification is present in all cases of Keutel syndrome and can involve the larynx, trachea, bronchi, nose and ears. The most consistent sign of calcification associated with this syndrome is abnormal cartilage calcification, that can be detected on routinely performed cephalograms ^26^.

In some cases, the linear ossification can mimic a foreign body. An ossified cricoid may be interpreted on lateral neck radiography as an ingested fishbone. Awareness of the existence of such ossification may reduce the number of unnecessary further upper airway examinations in patients suspected of having a foreign body ^27^. The most common foreign bodies impacted in the pharynx in adults are chicken and fish bones, while coins are the most common in children. The area located at the C6 level, posterior to the cricoid cartilage is the most common site of foreign body impaction ^3^. Rubin and Krost presented a case report of calcification in the thyroid cartilage in a 22-year-old woman, which was initially misdiagnosed as an aspirated tooth ^28^.

Lateral cephalograms taken before orthodontic treatment are able to provide a large number of radiographs taken in non-urgent situations and can, therefore, serve as a good tool for evaluating anatomical findings in the population. Good radiographic interpretation skills are essential for clinicians who use cephalometric radiographs to analyze craniofacial growth in orthodontics and maxillofacial surgery ^12^.

## Limitations

Radiographic examination is an accurate method for diagnosing and identifying both calcification and ossification, and in some cases can even distinguish between them ^10^. Although calcification and ossification of the laryngeal cartilage can be distinguished radiologically ^3^, the main purpose of this study was to find signs of mineralization; therefore, the terms calcification and ossification were used interchangeably, in accordance with the analysis performed in previous studies. Moreover, it was concluded that the degree of calcification is not a reliable indicator of age, with large inter- individual differences ^10^. Therefore, we did distinguish the calcifications into different types of laryngeal cartilages. The study sample comprises patients undergoing radiography for orthodontic reasons, and this could potentially limit the generalizability of our findings to a broader population. Factors not considered in our study, such as genetic, environmental, or lifestyle factors, could potentially influence the process of calcification. Our findings underscore the importance of a comprehensive clinical evaluation of an individual, moving beyond the sole reliance on the interpretation of radiographs. While our analysis may serve as a starting point for further research, it provides valuable clues and prompts additional questions regarding laryngeal health.

## Conclusions

Calcification can be detected by orthodontists trained in lateral cephalogram analysis and can be used as a screening or diagnostic tool to detect calcified areas in the larynx. The degree of calcification increased steadily, reaching 100% in women at age 30 and in men at age 50. In women, the degree of calcification was higher than in men from the age of 13 years and levelled off at the age of 50 years. The greatest differences in the degree of calcification by sex were found at ages 20–25 years, which was not observed in previous studies. Based on the results, effects of age and sex reliably determined the probability of laryngeal cartilage calcification.

Establishing clear guidelines for the clinical utilization of information related to laryngeal calcifications detected via lateral cephalometric radiographs requires careful consideration of the study's findings and their potential implications. Given the detection of laryngeal calcifications, collaboration with otolaryngologists (ENT specialists) is crucial. Laryngeal calcifications on lateral cephalometric radiographs should prompt a clinical assessment of the patient's laryngeal health. Patients exhibiting laryngeal calcifications along with symptoms such as hoarseness, difficulty swallowing, or other voice-related issues should undergo thorough investigation, including a detailed patient history, and subsequently be referred to an otolaryngologist for further examination and diagnostic tests.

To ensure comprehensive care, we recommend establishing a follow-up plan for patients with detected laryngeal calcifications to monitor symptom changes or the progression of the condition. Maintaining regular communication between orthodontic and otolaryngology teams can facilitate ongoing patient care. Additionally, considering the complexity of laryngeal structures, further imaging studies such as MRI may be proposed for a more detailed evaluation when necessary.

## Data Availability

The datasets used and/or analysed during the current study are available from the corresponding author on reasonable request.
